# Prostaglandins Differentially Regulate the Constitutive and Mechanosensitive Release of Soluble Nucleotidases in the Urinary Bladder Mucosa

**DOI:** 10.3390/ijms26010131

**Published:** 2024-12-27

**Authors:** Alejandro Gutierrez Cruz, Mahsa Borhani Peikani, Tori D. Beaulac, Violeta N. Mutafova-Yambolieva

**Affiliations:** Department of Physiology and Cell Biology, School of Medicine, University of Nevada Reno, Reno, NV 89557, USA

**Keywords:** nucleotidases, prostaglandins, ATP, bladder, urothelium, prostaglandin E_2_, prostaglandin D_2_, prostaglandin F_2α_

## Abstract

The urothelium and lamina propria (LP) contribute to sensations of bladder fullness by releasing multiple mediators, including prostaglandins (PGs) and adenosine 5′-triphosphate (ATP), that activate or modulate functions of cells throughout the bladder wall. Mediators that are simultaneously released in response to bladder distention likely influence each other’s mechanisms of release and action. This study investigated whether PGs could alter the extracellular hydrolysis of ATP by soluble nucleotidases (s-NTDs) released in the LP of nondistended or distended bladders. Using an ex vivo murine detrusor-free bladder model to access the LP during bladder filling and a sensitive HPLC-FLD detection methodology, we evaluated the decrease in ATP and the increase in adenosine 5′-diphosphate (ADP), adenosine 5′-monophosphate (AMP), and adenosine by s-NTDs released in the LP. Endogenous PGE_2_ increased the spontaneous but not the distention-induced release of s-NTD via EP2 and EP3 prostanoid receptors, whereas exogenous PGE_2_ increased the spontaneous s-NTD release via EP3, EP4, and FP receptors and the distention-induced s-NTD release via EP1-4 and FP receptors. Endogenous PGF_2α_, PGD_2_, and PGI_2_ did not change the s-NTD release. Exogenous PGD_2_ increased the spontaneous s-NTD release via DP2 receptors and the distention-induced s-NTD release via DP1 and DP2 receptors. Exogenous PGF_2α_ increased the spontaneous but not the distention-induced release of s-NTD via FP receptors. It is possible that higher concentrations of PGE_2_, PGF_2α_, and PGD_2_ (as expected in inflammation, bladder pain syndrome, or overactive bladder) potentiate the release of s-NTDs and the consecutive degradation of ATP as a safeguard mechanism to prevent the development of excessive bladder excitability and overactivity by high amounts of extracellular ATP.

## 1. Introduction

During filling of the bladder with urine, the bladder mucosa (urothelium and suburothelium/lamina propria, LP) detects bladder wall distention and releases signaling molecules such as acetylcholine, nitric oxide, neuropeptides, nerve growth factors, prostaglandins (PGs), and adenosine 5′-triphosphate (ATP) that affect the functions of cells throughout the bladder wall [1,2,3]. PGs, in particular, are eicosanoids synthesized through the oxidization of arachidonic acid by cyclooxygenase (COX) followed by the rapid conversion of a short-lived intermediary PGH_2_ to five different PGs (i.e., PGE_2_, PGD_2_, PGF_2α_, prostacyclin/PGI_2_, and thromboxane TXA_2_) by tissue-specific prostanoid synthases. PGs are released in all layers of the bladder wall in response to stretch, nerve stimulation, and chemical mediators [4,5,6,7,8], including ATP [9]. The production of PGs in healthy bladder tissues is relatively low and likely contributes to bladder homeostasis [10]. The synthesis and release of PGs can increase following injury or inflammation of the bladder mucosa [7,11,12]. High concentrations of extracellular PGs are pro-inflammatory and participate in the maintenance of chronic inflammation [13]. PGE_2_, PGD_2_, PGF_2α_, PGI_2_, and TXA_2_ are the five principal PGs that are identified in the bladder. PGs exert their physiological and pathophysiological functions by the activation of a number of prostanoid receptors such as EP1, EP2, EP3, EP4, FP, DP1, DP2, IP (I2), and TP (TXA_2_) receptors in membranes of cells throughout the bladder wall (reviewed in [14,15,16,17]). These receptors are coupled to various G protein subunits that stimulate various intracellular signaling pathways [18,19]. PGs participate in the regulation of micturition by affecting the relaxation and contraction of the bladder and the urethra [20]. Endogenous urothelial PGs appear to play a role in normal bladder physiology by maintaining optimum tone and contractility of the bladder and basal sensory output [21]. Exogenous PGs cause increased duration, frequency, and amplitude of bladder contractions; reduced voiding volume; and diminished functional capacity of the bladder [7,22,23,24].

Bladder distention during filling with urine also releases ATP from the bladder mucosa [25,26,27,28]. ATP activates P2X and P2Y receptors localized in the membranes of numerous cell types in the bladder wall. The activation of P2X2/X3 receptors on afferent neurons in the LP and urothelium, which trigger voiding reflexes, is believed to constitute a major physiological function of the urothelium-derived ATP [29,30]. Urothelial ATP can diffuse to the detrusor and cause muscle contraction. Therefore, ATP is a key regulator of bladder excitability. Systemic and local inflammation also increase urothelial release of ATP [31,32]. Notably, we have demonstrated that in addition to ATP, soluble nucleotidases (s-NTDs) are released in the LP [33] and lumen [34] of the bladder. The release of s-NTDs can occur spontaneously or in response to bladder distention, with the latter being more significant [33]. S-NTDs degrade sequentially ATP to ADP, AMP, and adenosine (ADO) [33,34]. Therefore, s-NTDs contribute to the mechanisms that determine the effective concentrations of ATP and metabolites at their receptor sites.

Collectively, the aforementioned studies suggest that under physiological or pathophysiological conditions, PGs, ATP, and s-NTDs are likely released simultaneously from the bladder mucosa and may affect each other’s actions deep in the bladder wall. Indeed, studies have shown that ATP can stimulate the synthesis and release of PGs in the bladder wall [9] and that exogenous PGs (e.g., PGE_2_) can increase the intravesical levels of ATP [6]. However, it is unknown whether exogenous PGs alter ATP levels because they affect ATP release or ATP metabolism by s-NTDs. The present study was designed to investigate the latter possibility. We aimed at addressing four questions: (1) Do PGE_2_, PGF_2α_, PGD_2_, PGI_2_, and TXA_2_ regulate the degradation of ATP by s-NTDs released in the bladder LP? (2) Is the degradation of ATP by s-NTDs influenced differently by endogenous and exogenous PGs? (3) Do PGs differentially alter the spontaneous and distention-induced release of s-NTDs? (4) What receptors mediate the effects of PGs on s-NTD release in the bladder LP, if any?

## 2. Results

### 2.1. Influences of Endogenous Activators of EP Prostanoid Receptors on Spontaneous Release of s-NTDs

**Figure 1:**
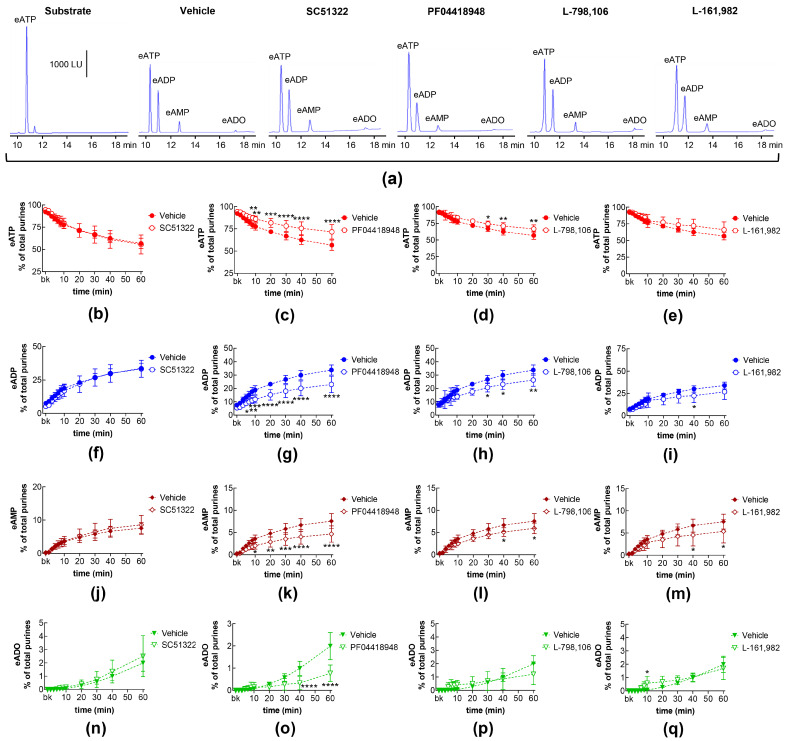
Effects of EP prostanoid receptor antagonists on the eATP hydrolysis by spontaneously released s-NTDs. Original HPLC chromatograms showing the hydrolysis of eATP and formation of eADP, eAMP, and eADO after 60 min of contact of the eATP substrate with s-NTDs released in EL solutions of nondistended bladder preparations treated with either vehicle (i.e., DMSO 0.2%) or EP receptor antagonists (**a**). The scale in the first panel applies to all chromatograms. LU, luminescence units. Summarized results demonstrating time courses of the eATP decrease (**b**–**e**) and the increase in eADP (**f**–**i**), eAMP (**j**–**m**), and eADO (**n**–**q**) in the presence of vehicle (n = 6) or of the EP1 antagonist SC51322 (1 μM, n = 6) (**b**,**f**,**g**,**n**), the EP2 receptor antagonist PF04418948 (1 μM, n = 7) (**c**,**g**,**k**,**o**), the EP3 receptor antagonist L-798,106 (0.25 μM, n = 5) (**d**,**h**,**l**,**p**), and the EP4 antagonist L-161,982 (1 μM, n = 6) (**e**,**i**,**m**,**q**). n, number of bladder preparations. Each purine is expressed as a percentage of the total amount of purines detected in EL solutions at each time point of enzymatic reaction. Asterisks denote significant differences from the vehicle control. * *p* < 0.05, ** *p* < 0.01, *** *p* < 0.001, **** *p* < 0.0001.

EP1, EP2, EP3, and EP4 receptors are expressed in the mouse bladder [35]. PGE_2_ is a primary activator of EP receptors [18] and is a dominant PG in the bladder [10]. To determine whether the activation of EP1-4 receptors by endogenously released PG(s) (e.g., PGE_2_) modulates the spontaneous release of s-NTDs in the LP, we evaluated the degradation of eATP to eADP, eAMP, and eADO in extraluminal (EL) solutions of nondistended bladders pretreated with selective antagonists of the four EP receptors. Table 1 shows the drugs and concentrations used in this study. As shown in Figure 1, the EP1 receptor selective antagonist SC51322 [36] had no effect on the degradation of eATP (i.e., the decrease in eATP and the increase in e-products) by spontaneously released s-NTDs (Figure 1a,b,f,j,n). In contrast, the EP2 receptor selective antagonist PF04418948 [37] significantly diminished the decrease in 1,*N*^6^-etheno-ATP (eATP) at 8–60 min of reaction and suppressed the increase in eADP (6–60 min), eAMP (10-60 min), and eADO (40–60 min) (Figure 1a,c,g,k,o). Reduced degradation of eATP to eADP and eAMP was also observed in the presence of the EP3 receptor selective antagonist L-798,106 [38] at 30–60 min of reaction (Figure 1a,d,h,l,p). The EP4 receptor selective antagonist L-161,982 [39] tended to diminish the eATP degradation, which reached statistical significance in the levels of eAMP at 40 and 60 min of reaction and at single time points of eADP and eADO formation (Figure 1a,e,i,m,q). These results suggest that PGs are likely secreted at rest in the bladder LP and could facilitate the spontaneous release of s-NTDs by activation of EP2 and EP3 prostanoid receptors.

### 2.2. Influences of Endogenous Activators of EP Prostanoid Receptors on Distention-Induced Release of s-NTDs

**Figure 2:**
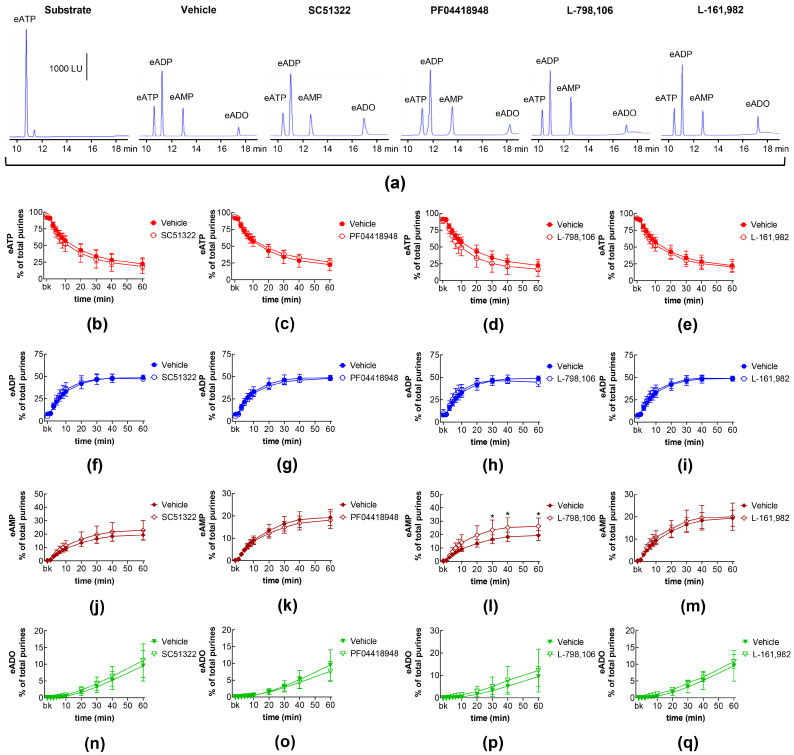
Effects of EP prostanoid receptor antagonists on the eATP hydrolysis by s-NTDs released during distention of the bladder wall. Original HPLC chromatograms showing the hydrolysis of eATP and the formation of eADP, eAMP, and eADO after 60 min of contact of the eATP substrate with s-NTDs released in EL solutions of distended bladder preparations treated with either vehicle (i.e., DMSO 0.2%) or EP receptor antagonists (**a**). The scale in the first panel applies to all chromatograms. LU, luminescence units. Summarized results demonstrating time courses of the eATP decrease (**b**–**e**) and the increase in eADP (**f**–**i**), eAMP (**j**–**m**), and eADO (**n**–**q**) in the presence of vehicle (n = 6) or the EP1 antagonist SC51322 (1 μM, n = 6) (**b**,**f**,**g**,**n**), the EP2 receptor antagonist PF04418948 (1 μM, n = 7) (**c**,**g**,**k**,**o**), the EP3 receptor antagonist L-798,106 (0.25 μM, n = 5) (**d**,**h**,**l**,**p**), and the EP4 antagonist L-161,982 (1 μM, n = 6) (**e**,**i**,**m**,**q**). n, number of bladder preparations. Each purine is expressed as a percentage of the total amount of purines detected in EL solutions at each time point of enzymatic reaction. Asterisks denote significant differences from the vehicle control. * *p* < 0.05.

The degradation of eATP to eADP, eAMP, and eADO by s-NTDs released upon distention of the LP during bladder filling remained unaffected in the presence of antagonists of EP1 (SC51322; Figure 2a,b,f,j,n), EP2 (PF04418948; Figure 2a,c,g,k,o), and EP4 (L-161,982; Figure 2a,e,i,m,q) prostanoid receptors. Likewise, the decrease in eATP and the increase in eADP and eADO remained unaffected in the presence of the EP3 receptor antagonist L-798,106 (Figure 2a,d,h,p). However, the amounts of eAMP were increased at 30–60 min of enzymatic reaction (Figure 2a,l). These results suggest that the activation of EP1, EP2, and EP4 receptors by endogenous PGs does not regulate the distention-induced release of s-NTDs and the consequent degradation of extracellular ATP. However, EP3 receptors may play a role in regulating the conversion of AMP to ADO during ATP degradation.

### 2.3. Influences of Endogenous Activators of Prostanoid DP and FP Receptors on Spontaneous and Distention-Induced Release of s-NTDs

**Figure 3:**
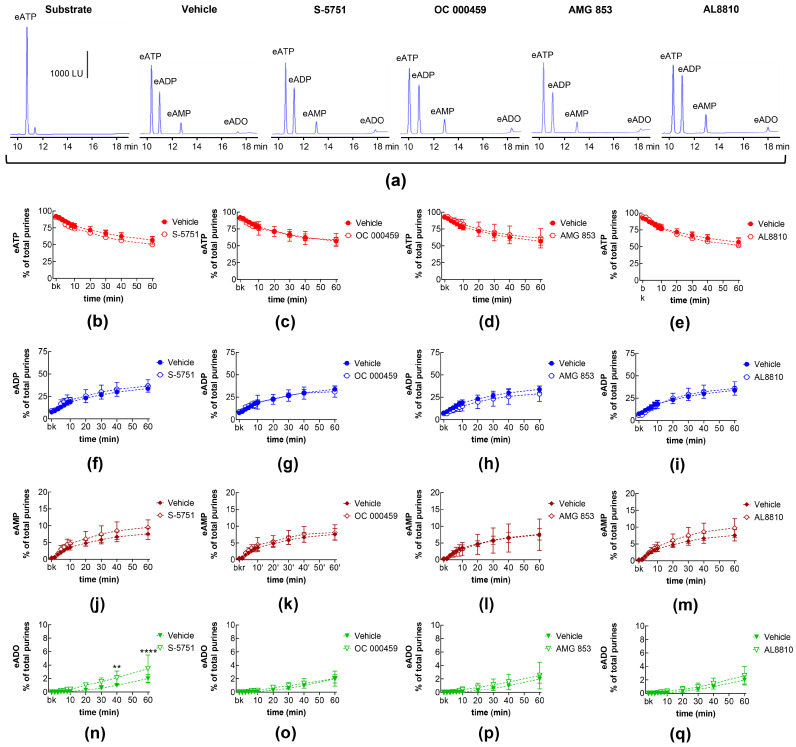
Effects of DP and FP prostanoid receptor antagonists on the eATP hydrolysis by spontaneously released s-NTDs. Original HPLC chromatograms showing the hydrolysis of eATP and formation of eADP, eAMP, and eADO after 60 min of contact of the eATP substrate with s-NTDs released in EL solutions of nondistended bladder preparations treated with either vehicle (i.e., DMSO 0.2%) or receptor antagonists (**a**). The scale in the first panel applies to all chromatograms. LU, luminescence units. Summarized results demonstrating time courses of the eATP decrease (**b**–**e**) and the increase in eADP (**f**–**i**), eAMP (**j**–**m**), and eADO (**n**–**q**) in the presence of vehicle (n = 6) or the DP1 antagonist S-5751 (1 μM, n = 4) (**b**,**f**,**g**,**n**), the DP2 receptor antagonist OC000459 (10 μM, n = 4) (**c**,**g**,**k**,**o**), the CRTH2/DP receptor antagonist AMG 853 (1 μM, n = 8) (**d**,**h**,**l**,**p**), and the FP receptor antagonist AL8810 (10 μM, n = 6) (**e**,**i**,**m**,**q**). n, number of bladder preparations. Each purine is expressed as a percentage of the total amount of purines detected in EL solutions at each time point of enzymatic reaction. Asterisks denote significant differences from the vehicle control. ** *p* < 0.01, **** *p* < 0.0001.

**Figure 4:**
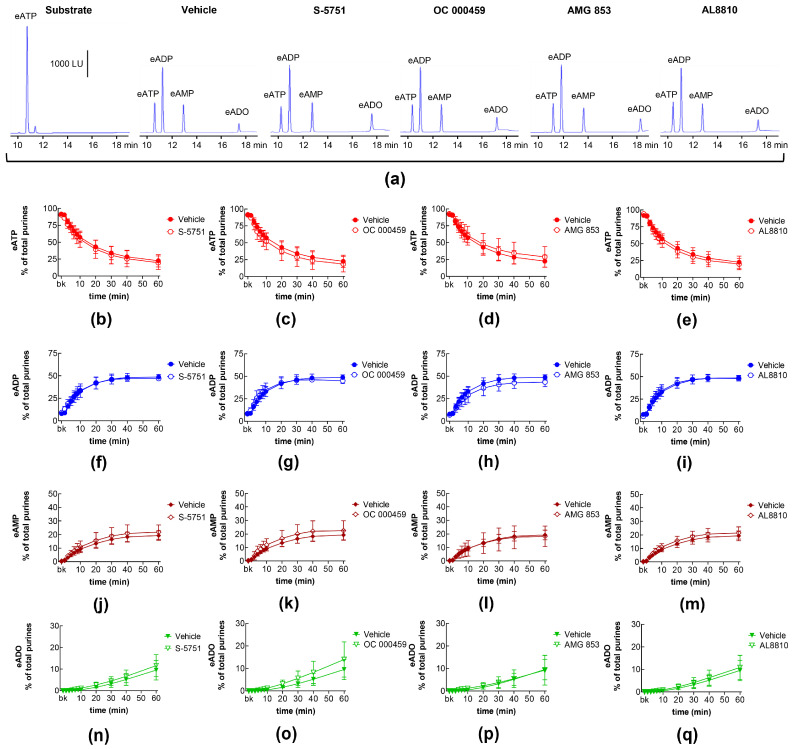
Effects of DP and FP prostanoid receptor antagonists on the eATP hydrolysis by s-NTDs released during distention of the bladder wall. Original HPLC chromatograms showing the hydrolysis of eATP and formation of eADP, eAMP, and eADO after 60 min of contact of the eATP substrate with s-NTDs released in EL solutions of distended bladder preparations treated with either vehicle (i.e., DMSO 0.2%) or receptor antagonists (**a**). The scale in the first panel applies to all chromatograms. LU, luminescence units. Summarized results demonstrating time courses of the eATP decrease (**b**–**e**) and the increase in eADP (**f**–**i**), eAMP (**j**–**m**), and eADO (**n**–**q**) in the presence of vehicle (n = 6) or the DP1 antagonist S-5751 (1 μM, n = 4) (**b**,**f**,**g**,**n**), the DP2 receptor antagonist OC000459 (10 μM, n = 4) (**c**,**g**,**k**,**o**), the DP1/DP2 receptor antagonist AMG 853 (1 μM, n = 8) (**d**,**h**,**l**,**p**), and the FP receptor antagonist AL8810 (10 μM, n = 6) (**e**,**i**,**m**,**q**). n, number of bladder preparations. Each purine is expressed as a percentage of the total amount of purines detected in EL solutions at each time point of enzymatic reaction.

DP receptors are found within the detrusor and bladder mucosa [40]. PGD_2_ is the primary endogenous ligand of these receptors [16], and it is found released in the bladder urothelium [41]. The degradation of eATP and the formation of e-products remained unchanged in the presence of the selective DP1 receptor antagonist S-5751 [42], the selective DP2 receptor antagonist OC 000459 [43], and the dual DP1 and DP2 receptor antagonist AMG 853 [44] (Figure 3). Only the eADO increase reached statistical significance at 40–60 min of reaction in EL solutions of nondistended bladders treated with S-5751 (Figure 3n). The selective FP receptor antagonist AL8810 [45] had no effect on the eATP decrease and the increase in eADP, eAMP, and eADO in EL solutions collected from both nondistended (Figure 3) and distended (Figure 4) bladder preparations.

### 2.4. Influences of Endogenous Activators of Prostanoid IP Receptors on Spontaneous and Distention-Induced Release of s-NTDs

The selective IP receptor antagonist RO1138452 [46] did not alter the decrease in eATP and the increase in eADP, eAMP, and eADO in EL solutions of nondistended bladders (Table 2). In EL solutions from distended bladder preparations, the eADO levels at 60 min of reaction were significantly lower in the bladders treated with RO1113842 compared with those treated with the vehicle (Table 3).

### 2.5. Effects of COX Inhibition on Spontaneous and Distention-Induced Release of s-NTDs

The decrease in eATP and increase in eADP and eAMP in EL solutions of both nondistended and distended bladder preparations remained unaltered by treating the preparations with the COX1/COX2 inhibitor indomethacin (data at 60 min of reaction shown in Table 2 and Table 3). However, the increase in eADO was significantly diminished in the EL solutions of both nondistended and distended bladders treated with indomethacin (Table 2 and Table 3).

### 2.6. Effects of Exogenous PGI_2_ and TXA_2_ Analog on the Spontaneous and Distention-Induced Release of s-NTDs

The degradation of eATP in EL solutions collected from nondistended and distended preparations treated with either PGI2 or U46619 (a stable analog of TXA_2_) [47] remained unchanged compared with vehicle controls (Table 2 and Table 3). Likewise, the application of PGI_2_ to the organ chambers with bladder preparations pretreated with the IP receptor antagonist RO1138452 had no significant effect on the eATP hydrolysis. The levels of eATP substrate and e-products at the ends of the enzymatic reactions (60 min) were similar in vehicle controls and drug treatments (Table 2 and Table 3).

### 2.7. Effects of Exogenous PGE_2_ on the Spontaneous Release of s-NTDs and Involvement of EP Prostanoid Receptors

**Figure 5:**
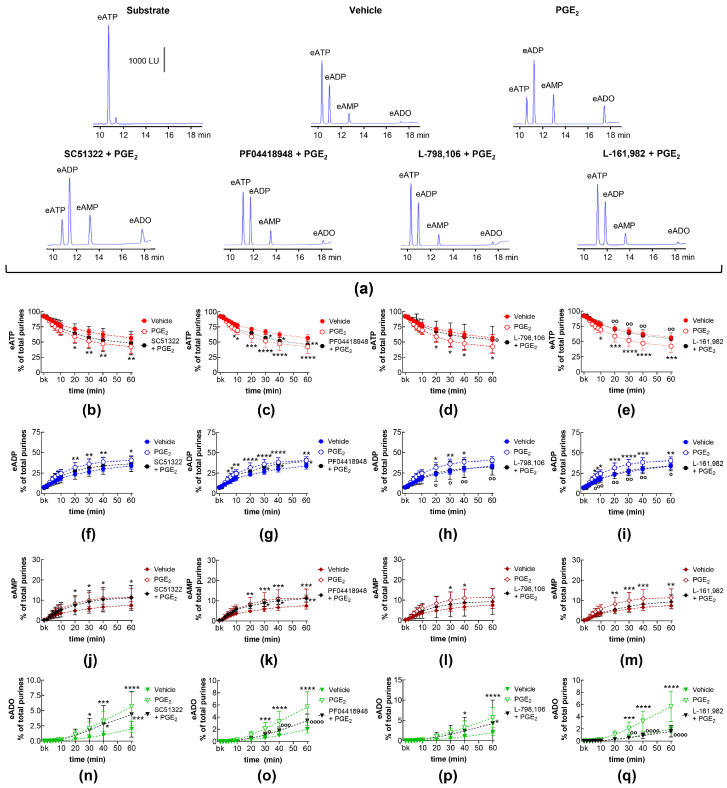
eATP hydrolysis by s-NTDs released in EL solutions of nondistended bladder preparations treated with exogenous PGE_2_ in the absence or presence of EP prostanoid receptor antagonists. Original HPLC chromatograms showing the eATP degradation after 60 min of contact with the EL solutions (**a**). The scale in the first panel applies to all chromatograms. LU, luminescence units. Summarized results demonstrating time courses of the eATP decrease (**b**–**e**) and the increase in eADP (**f**–**i**), eAMP (**j**–**m**), and eADO (**n**–**q**) by PGE2 (10 μM) in the presence of vehicle (n = 8) or of the EP1 antagonist SC51322 (1 μM, n = 6) (**b**,**f**,**g**,**n**), the EP2 receptor antagonist PF04418948 (1 μM, n = 5) (**c**,**g**,**k**,**o**), the EP3 receptor antagonist L-798,106 (0.25 μM, n = 9) (**d**,**h**,**l**,**p**), and the EP4 antagonist L-161,982 (1 μM, n = 4) (**e**,**i**,**m**,**q**). n, number of bladder preparations. Asterisks denote significant differences from the vehicle control (n = 6). * *p* < 0.05, ** *p* < 0.01, *** *p* < 0.001, **** *p* < 0.0001. Open circles denote significant differences of eATP degradation in PGE_2_ alone vs. EP receptor antagonist + PGE_2_. ^o^ *p* < 0.05, ^oo^ *p* < 0.01, ^ooo^ *p* < 0.001, ^oooo^ *p* < 0.0001. Two-way ANOVA with Tukey’s multiple comparisons test.

Next, we investigated whether exogenous PGE_2_ affected the degradation of eATP by s-NTDs in the bladder LP. As shown in Figure 5, the treatment of the LP with PGE_2_ (10 μM) led to an accelerated decrease in eATP substrate at 20–60 min of reaction and an increase in the eATP products eADP (20–60 min), eAMP (20–60 min), and eADO (30–60 min) in EL solutions of nondistended bladder preparations. The effect of PGE_2_ was largely unaffected by the EP1 receptor antagonist SC51322 (Figure 5a,b,f,j,n) or by the EP2 receptor antagonist PF04418948 (Figure 5a,c,g,k). However, PF04418948 significantly diminished the eADO amounts at 30–60 min of reaction (Figure 5o). In contrast, the increasing effect of PGE_2_ on the eATP degradation was abolished by L-798,106 (Figure 5a,d,h,l,p) and L-161,982 (Figure 5a,e,i,m,q), antagonists of EP3 and EP4 receptors, respectively. These results suggest that exogenous PGE_2_ increases the spontaneous release of s-NTDs via activation of EP3 and EP4 but not EP1 and EP2 prostanoid receptors.

### 2.8. Effects of Exogenous PGE_2_ on Distention-Induced Release of s-NTDs and Involvement of EP Prostanoid Receptors

**Figure 6:**
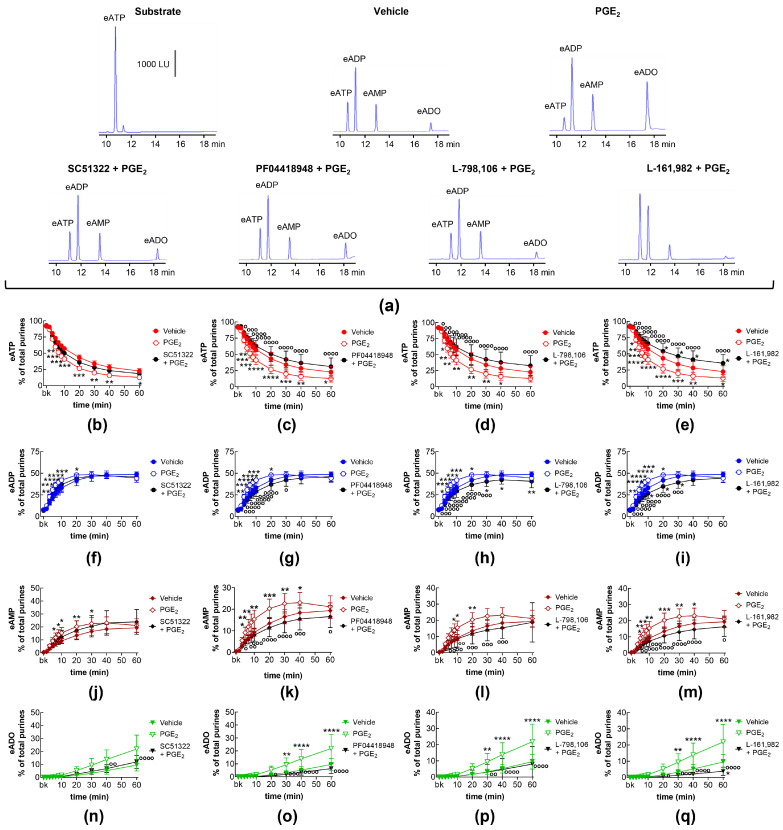
eATP hydrolysis by s-NTDs released in EL solutions of distended bladder preparations treated with exogenous PGE_2_ in the absence or presence of EP prostanoid receptor antagonists. Original HPLC chromatograms showing the eATP degradation after 60 min of contact with the EL solutions (**a**). The scale in the first panel applies to all chromatograms. LU, luminescence units. Summarized results demonstrating time courses of the eATP decrease (**b**–**e**) and the increase in eADP (**f**–**i**), eAMP (**j**–**m**), and eADO (**n**–**q**) by PGE2 (10 μM) in the presence of vehicle (n = 8) or of the EP1 antagonist SC51322 (1 μM, n = 5) (**b**,**f**,**g**,**n**), the EP2 receptor antagonist PF04418948 (1 μM, n = 5) (**c**,**g**,**k**,**o**), the EP3 receptor antagonist L-798,106 (0.25 μM, n = 9) (**d**,**h**,**l**,**p**), and the EP4 antagonist L-161,982 (1 μM, n = 4) (**e**,**i**,**m**,**q**). n, number of bladder preparations. Asterisks denote significant differences from the vehicle control (n = 6). * *p* < 0.05, ** *p* < 0.01, *** *p* < 0.001, **** *p* < 0.0001. Open circles denote significant differences of eATP degradation in PGE_2_ alone vs. EP receptor antagonist + PGE_2_. ^o^ *p* < 0.05, ^oo^ *p* < 0.01, ^ooo^ *p* < 0.001, ^oooo^ *p* < 0.0001. Two-way ANOVA with Tukey’s multiple comparisons test.

Exogenous PGE_2_ also increased the degradation of eATP in EL solutions collected from distended bladder preparation during bladder filling (Figure 6). The effect of PGE_2_ was largely inhibited by pretreatment of the bladder preparations with SC51322 (Figure 6a,b,f,j,n), PF04418948 (Figure 6a,c,g,k,o), and L-798,106 (Figure 6a,d,h,l,p). Interestingly, in the presence of L-106,982, the potentiating effect of exogenous PGE_2_ on eATP degradation was inverted to inhibition of the eATP decrease and e-product increase (Figure 6a,e,i,m,q).

### 2.9. Effects of FP Receptor Blockade on the Effects of Exogenous PGE_2_ and PGF_2α_ on Spontaneous and Distention-Induced Release of s-NTDs

**Figure 7:**
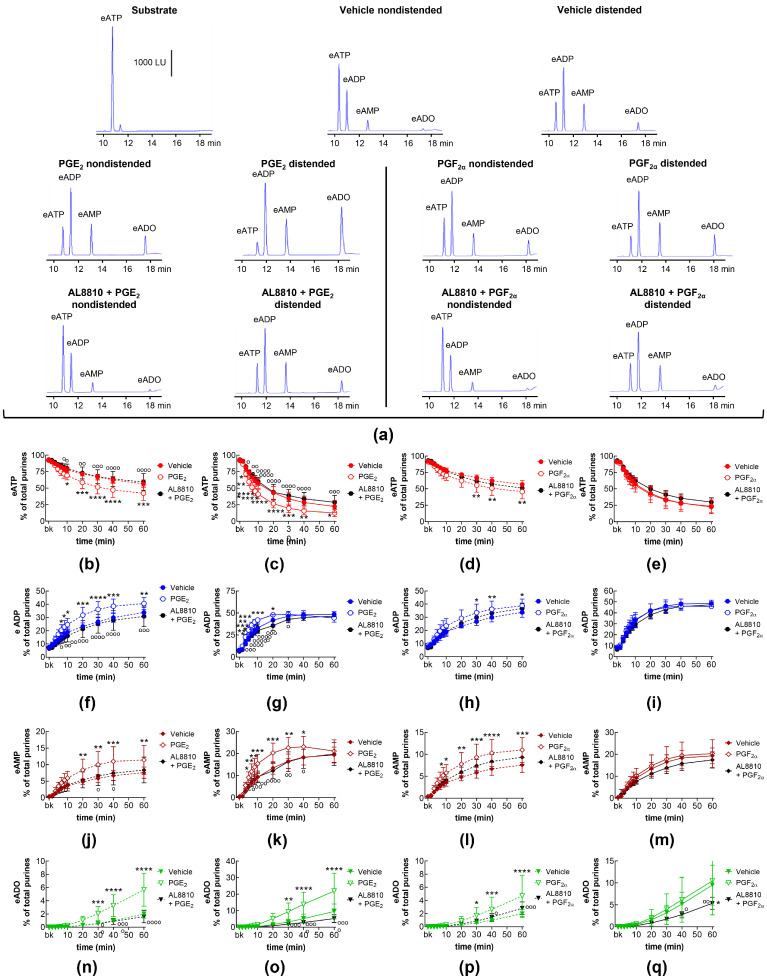
eATP hydrolysis by s-NTDs released in EL solutions of nondistended or distended bladder preparations treated with exogenous PGE_2_ or PGF_2α_ in the absence or presence of an FP prostanoid receptor antagonist. Original chromatograms showing the eATP degradation after 60 min of contact with the EL solutions of nondistended and distended bladders (**a**). The scale in the first panel applies to all chromatograms. LU, luminescence units. Summarized results demonstrating time courses of the eATP decrease and the increase in eADP, eAMP, and eADO in EL solutions of nondistended (**b**,**f**,**j**,**n**) or distended (**c**,**g**,**k**,**o**) bladders treated with PGE_2_ (n = 8) or AL8810 + PGE2 (n = 4). Effects of PGF_2α_ (n = 6) or AL8810 + PGF_2α_ (n = 4) on time courses of eATP hydrolysis to eADP, eAMP, and eADO in EL solutions of nondistended (**d**,**h**,**l**,**p**) or distended (**e**,**i**,**m**,**q**) bladders are shown in the panels to the right of the solid black line. n, number of bladder preparations. Asterisks denote significant differences vs. the vehicle controls (n = 6). * *p* < 0.05, ** *p* < 0.01, *** *p* < 0.001, **** *p* < 0.0001. Open circles denote significant differences of eATP degradation in PG alone vs. AL8810 + PG. ^o^ *p* < 0.05, ^oo^ *p* < 0.01, ^ooo^ *p* < 0.001, ^oooo^ *p* < 0.0001. Two-way ANOVA with Tukey’s multiple comparisons test.

In addition to EP receptors, PGE_2_ has been shown to stimulate FP prostanoid receptors [24]. In this study, the selective FP receptor inhibitor AL8810 abolished the increasing effect of exogenous PGE_2_ on eATP degradation in EL solutions collected from nondistended (Figure 7a,b,f,j,n) and from distended (Figure 7a,c,g,k,o) preparations.

PGF_2α_ is assumed to be the primary endogenous ligand of the FP receptor [19,36]. In the present study, exogenous PGF_2α_ increased the degradation of eATP in the EL solutions of nondistended bladders, and this effect of PGF_2α_ was inhibited by AL8810 (Figure 7a,d,h,l,p). The degradation of eATP and the formation of eADP and eAMP in EL solutions of distended preparations were not affected by AL8810 (Figure 7a,e,i,m,q). Interestingly, in the presence of AL8810, exogenous PGF_2α_ did not affect the eATP degradation or the increase in eADP and eAMP (Figure 7e,i,m), but it reduced the formation of eADO (Figure 7q).

### 2.10. Effects of Exogenous PGD_2_ on Spontaneous Release of s-NTDs and Involvement of DP Prostanoid Receptors

**Figure 8:**
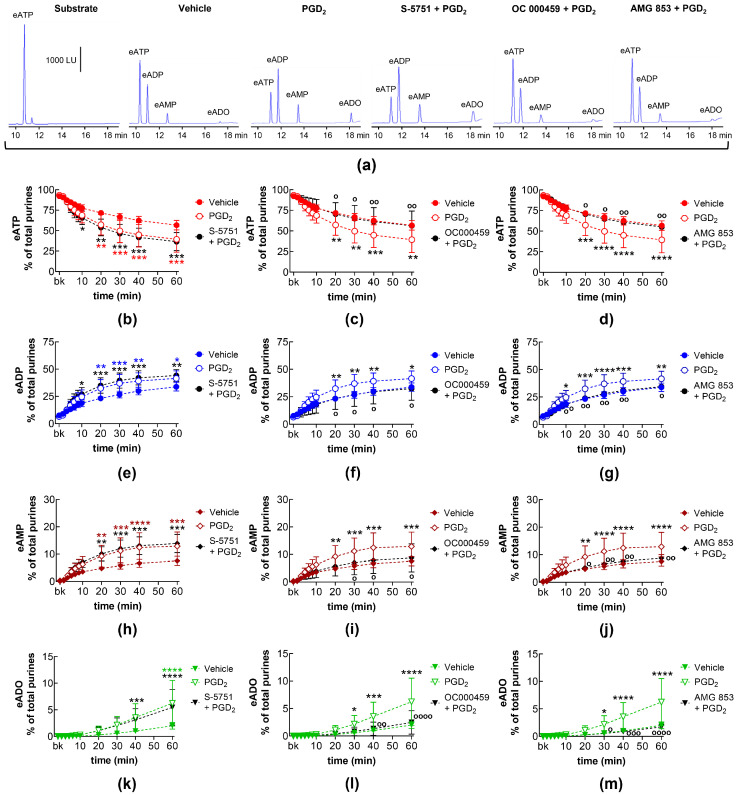
eATP hydrolysis by s-NTDs released in EL solutions of nondistended bladder preparations treated with exogenous PGD_2_ in the absence or presence of DP prostanoid receptor antagonists. Original HPLC chromatograms showing the eATP degradation after 60 min of contact with the EL solutions (**a**). The scale in the first panel applies to all chromatograms. LU, luminescence units. Summarized results demonstrating time courses of the eATP decrease (**b**–**d**) and the increase in eADP (**e**–**g**), eAMP (**h**–**j**), and eADO (**k**–**m**) by PGD2 (10 μM, n = 7) in the presence of vehicle or of the DP1 antagonist S-5751 (1 μM, n = 4) (**b**,**e**,**h**,**k**), the DP2 receptor antagonist OC000459 (10 μM, n = 4) (**c**,**f**,**i**,**l**), and the dual CRTH2/DP receptor antagonist AMG 853 (1 μM, n = 4) (**d**,**g**,**j**,**m**). n, number of bladder preparations. Asterisks denote significant difference from the vehicle control (n = 6). Black asterisks denote significant differences of PGD_2_ effects from the vehicle control; colored asterisks denote significant differences of DP receptor antagonist + PGD_2_ from the vehicle control. * *p* < 0.05, ** *p* < 0.01, *** *p* < 0.001, **** *p* < 0.0001. Open circles denote significant differences of eATP degradation in PGD_2_ alone vs. DP receptor antagonist + PGD_2_. ^o^ *p* < 0.05, ^oo^ *p* < 0.01, ^ooo^ *p* < 0.001, ^oooo^ *p* < 0.0001. Two-way ANOVA with Tukey’s multiple comparisons test.

In EL solutions collected from nondistended bladder preparations, exogenous PGD_2_ increased the degradation of eATP (10–60 min) and the formation of eADP (10–60 min), eAMP (10–60 min), and eADO (40–60 min) (Figure 8). The increasing effect of PGD_2_ on eATP hydrolysis was not affected by the DP1 receptor selective antagonist S-5751 (Figure 8a,b,e,h,k) but was inhibited in the presence of the DP2 receptor selective antagonist OC000459 (Figure 8a,c,f,i,l) and the DP1/DP2 antagonist AMG 853 (Figure 8a,d,g,j,m).

### 2.11. Effects of Exogenous PGD_2_ on Distention-Induced Release of s-NTDs and Involvement of DP Prostanoid Receptors

**Figure 9:**
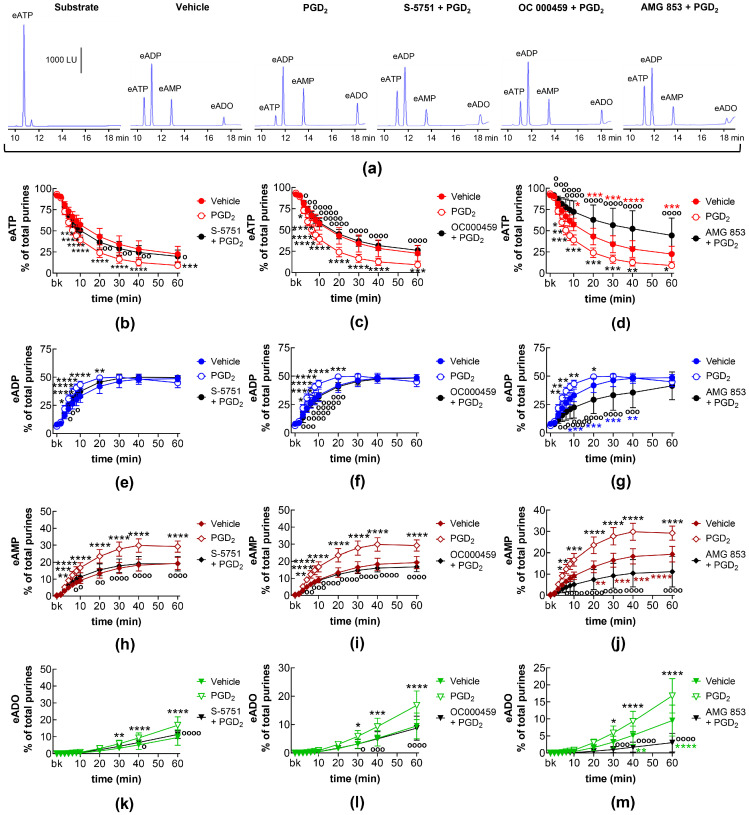
eATP hydrolysis by s-NTDs released in EL solutions of distended bladder preparations treated with exogenous PGD_2_ in the absence or presence of DP prostanoid receptor antagonists. Original HPLC chromatograms showing the eATP degradation after 60 min of contact with the EL solutions (**a**). The scale in the first panel applies to all chromatograms. LU, luminescence units. Summarized results demonstrating time courses of the eATP decrease (**b**–**d**) and the increase in eADP (**e**–**g**), eAMP (**h**–**j**), and eADO (**k**–**m**) by PGD2 (10 μM, n = 7) in the presence of vehicle or of the DP1 antagonist S-5751 (1 μM, n = 4) (**b**,**e**,**h**,**k**), the DP2 receptor antagonist OC000459 (10 μM, n = 4) (**c**,**f**,**i**,**l**), and the dual CRTH2/DP receptor antagonist AMG 853 (1 μM, n = 4) (**d**,**g**,**j**,**m**). n, number of bladder preparations. Asterisks denote significant differences from the vehicle control (n = 6). Black asterisks denote significant differences of PGD_2_ effects from the vehicle control; colored (red, blue, brown, green) asterisks denote significant difference of DP receptor antagonist + PGD_2_ from the vehicle control. * *p* < 0.05, ** *p* < 0.01, *** *p* < 0.001, **** *p* < 0.0001. Open circles denote significant differences of eATP degradation in PGD_2_ alone vs. DP receptor antagonist + PGD_2_. ^o^ *p* < 0.05, ^oo^ *p* < 0.01, ^ooo^ *p* < 0.001, ^oooo^ *p* < 0.0001. Two-way ANOVA with Tukey’s multiple comparisons test.

In EL solutions of distended bladder preparations treated with PGD_2_, the decrease in eATP (10–60 min) and the increase in eADP (10–60 min), eAMP (20–60 min), and eADO (40–60 min) was potentiated (Figure 9). The increasing effect of PGD_2_ on eATP degradation was abolished in the presence of the selective DP1 receptor antagonist S-5751 (Figure 9a,b,e,h,k) and the DP2 selective antagonist OC000459 (Figure 9a,c,f,i,l). In the presence of the dual DP1/DP2 receptor antagonist AMG 853, the effect of PGD_2_ was inverted to inhibition of eATP hydrolysis (Figure 9a,d,g,j,m).

## 3. Discussion

The present study provides the first evidence for modulation of the extracellular ATP metabolism in the bladder LP. The main findings were that (1) both spontaneous and mechanosensitive release of s-NTDs and the consequent degradation of ATP were regulated by different PG-mediated mechanisms, and (2) endogenous and exogenous PGs differentially influenced the release of s-NTDs in the bladder LP.

The bladder urothelium plays a central role in sensing the filling state of the bladder and communicating this information to cells within the bladder wall and to the CNS [48,49]. These processes involve the release of chemical mediators such as acetylcholine [50,51], nitric oxide [52], neuropeptides [53], bradykinin [54], neurotrophins [55], prostaglandins [11,55], purines (i.e., ATP [28], and metabolic enzymes [33,34]). Since many of these mediators are released simultaneously at rest or in response to the same stimuli (e.g., stretch of the bladder wall during bladder filling), it is possible that released mediators not only act on their specific cell/receptor/ion channel targets but may also interfere with each other’s release and/or action. Such influences might occur under normal physiological conditions or may become particularly prominent in pathophysiological states like inflammation or non-inflammatory pathologies associated with bladder activity disorders [48]. Thus, understanding the mechanisms by which these mediators interact to regulate bladder function could put forward potential therapeutic targets for treating bladder excitability disorders. Studies have suggested that stretch-induced release of ATP can be augmented by bradykinin [56] or that ATP can induce release of urothelial acetylcholine [57]. Notably, however, possible impacts of mediators on each other’s metabolism remain severely understudied.

Upon release, ATP exerts its biological activity by activating P2X and P2Y purinergic receptors present in different cell types throughout the bladder wall and is essential for initiating the voiding reflex and for maintaining proper bladder excitability [30]. Once released, ATP is degraded sequentially to ADP, AMP, and ADO by a large number of membrane-bound and soluble/releasable NTDs that perform different steps of the extracellular ATP degradation [33,58]. Some of the ATP metabolites (i.e., ADP and ADO) have their own biological activity and can also affect bladder excitability. While both ATP and ADP are considered excitatory mediators in terms of detrusor contractility and sensory neuron activity, ADO is considered to be an inhibitory mediator that suppresses detrusor contractility and ATP release from efferent parasympathetic neurons [30]. Therefore, understanding the mechanisms that regulate the extracellular metabolism of ATP is important for the effort to control bladder excitability. Membrane-bound NTDs are ubiquitously expressed and therefore difficult to target specifically. The release of s-NTDs might be more localized and provide more precise targets for modulation of the effective concentrations of ATP at its receptor sites. We determined that the release of s-NTDs in the bladder LP was a complex, highly regulated mechanosensitive process that involved PIEZO channels, PAC1 receptors, P2X7 receptors, and pannexin 1 channels [59,60]. We also found that endogenous and exogenous CGRP, substance P, and PACAP38 differentially modulated the spontaneous and distention-induced release of s-NTDs in the LP [61]. Such interactions may be particularly important in bladder dysfunctions caused by inflammation that is characterized by increased release of both neuropeptides [62] and ATP [63]. PGs are local mediators that also participate in the development and maintenance of chronic inflammation and other bladder disorders [7,13]. Studies have proposed that ATP promotes PG synthesis and PG release from the detrusor muscle and the bladder urothelium and LP [30,64] or that, conversely, PGs modulate the release of ATP [7]. However, changes in the amounts of ATP in tissue fluids could result from altered ATP release, altered ATP hydrolysis, or both. To the best of our knowledge, it is currently unknown whether PGs modulate the degradation of extracellular ATP in physiological or pathophysiological conditions.

In the present study, we investigated potential influences of endogenous and exogenous PGs on the degradation of ATP to ADP, AMP, and ADO by recently discovered s-NTDs [33] that are released from the urothelium/LP of unfilled/nondistended and filled/distended mouse bladders. Additionally, we explored the potential role of different receptor targets for PGs on the extracellular hydrolysis of ATP by s-NTDs released in the LP at rest and during bladder filling. As in previous studies, we carried out these investigations in an ex vivo mouse bladder model with intact bladder mucosa but without the detrusor, which enabled direct access to the suburothelial surface of the bladder mucosa during authentic bladder filling. This model was particularly suitable for studying *local* mechanisms of regulation in the urothelium and LP without influences of the detrusor muscle, systemic circulation, and the CNS [65]. We also used 1,*N*^6^-etheno-ATP (eATP) as a substrate to increase the specificity, sensitivity, and accuracy of monitoring substrate decrease and product increase by enzymes that were released in tissue bathing solutions [33,59,60,61]. It has been confirmed that NTDs metabolize etheno-derivatized nucleotides similarly to their non-derivatized counterparts [66].

PG-synthesizing enzymes (e.g., COX-1 and COX-2), as well as the five main PGs, PGE_2_, PGF_2a_, PGD_2_, PGI_2_, and TXA_2_, and their receptors, are all expressed in the bladders of different species [7,23]. PGs are synthesized in both the bladder urothelium with the lamina propria and in the detrusor smooth muscle. In the bladder mucosa, constitutive COX-1 is expressed predominantly in the basal and intermediate layers of the urothelium and in cells in the LP, indicating that the bladder mucosa is capable of producing PGs required for the maintenance of homeostasis [7]. PGs are released by stretch, nerve stimulation, or activation with other mediators [7]. They are involved in several processes in the urinary bladder, including modulation of detrusor muscle tone, release of sensory mediators from the urothelium, pain sensation, and inflammation [7]. Augmented urothelial release of PGs is observed in animals and humans with overactive bladders and in response to inflammation [67]. The released PGs activate a panel of G-protein coupled receptors [16,18,19] and regulate the detrusor tone and contractility and the activity of sensory neurons within the bladder wall [21,68]. Each type of PG can activate multiple PG receptors, but their binding affinities vary significantly. PGE_2_ has a higher affinity for the four EP receptors (EP1–EP4), while PGF_2α_ has a higher affinity for FP receptors. PGD_2_ binds preferentially to DP1 and DP2 receptors, while IP receptors primarily respond to PGI_2_, and TP receptors are activated by TXA_2_ [16]. However, each PG type could activate multiple receptors albeit with different strength.

To determine whether endogenous PGs regulated the release of s-NTDs, we first applied selective PG receptor antagonists and then measured the hydrolysis of eATP in EL solutions of unfilled and filled bladders. Antagonists of EP1, EP4, DP1, DP2, and FP prostanoid receptors did not change the basal or the distention-induced release of s-NTDs in the LP, suggesting that endogenous activators of these receptors may not be necessary for the control of s-NTD release. The lack of effect of these receptor antagonists could be due to (i) low expression of the corresponding receptors in the mouse bladder urothelium/LP, (ii) insufficient concentrations of PGs (e.g., PGE2, PGD_2_, and PGF_2α_) to activate the receptors, or (iii) an inability of these receptors to control the s-NTD release. Another possibility could be that the receptor antagonists were used in subthreshold concentrations unable to effectively block the receptor targets. This was highly unlikely, however, since the same antagonists at the same concentrations were able to inhibit the effects of exogenously applied PGs (discussed below). PG production is generally very low in healthy (e.g., uninflamed) tissues [69], including the bladder [10], leading to diminished efficacy of receptor activation by endogenous PGs. In agreement with this, the treatment of bladders with the COX inhibitor indomethacin led to a slightly reduced eATP degradation by s-NTDs that was manifested by diminished eADO production but unaltered eATP decrease and eADP and eAMP increase. In contrast to the observations described above, the basal release of s-NTDs and the consequent degradation of eATP were diminished in the presence of EP2 and EP3 receptor antagonists, suggesting that endogenous PGE_2_ stimulated release of s-NTDs by activating EP2 and EP3 receptors on cells in the LP. The EP3 receptor blockade had a weaker effect on the spontaneous s-NTD release than the blockade of EP2 receptors. It was interesting that inhibition of two receptors that were coupled to opposing signaling pathways elicited qualitatively similar effects on the basal s-NTD release. The EP2 receptor couples to the Gα_s_ subunit that activates the adenylate cyclase to generate cAMP, whereas the EP3 receptor couples to the Gα_i_ subunit that downregulates the adenylate cyclase–cAMP signaling [16]. In effect, however, prostanoid receptor mediated mechanisms are more complex than mere activation of the main G subunit that is ascribed to a particular receptor type. PG receptors have numerous isoforms that could be coupled to multiple different G subunits [16,70]. For example, the mouse EP3 receptor has three isoforms—α, β, and γ—that couple to G_12/13_ to activate the Rho pathway for smooth muscle contraction. EP3γ also couples to Gα_s_. Consequently, activation of the EP3 receptor with PGE_2_ can activate signaling pathways that are associated with Gα_i_, G_12/13_, and Gα_s_ depending on the cellular context [17,70]. Such an ability to couple to multiple G subunits and signaling pathways is characteristic for the majority of prostanoid receptors [16,17]. Due to a range of intracellular signaling pathways that mediate the effects of PG receptor activation on cell function, it is challenging to assign specific signaling mechanisms to the modulating effects of endogenous PGs on the spontaneous release of s-NTDs.

The distention-induced release of s-NTDs and consequent degradation of eATP in EL solutions of bladders filled at a physiological filling rate were largely unaffected by all EP receptor antagonists with the exception of increased eAMP at 30–60 min of enzymatic reaction in the presence of the EP3 receptor antagonist. This effect could be due to diminished release of soluble 5′-nucleotidase (NT5E) or alkaline phosphatase (ALPL), both of which are released in the LP of the mouse bladder [33] and can convert eAMP to eADO [33,58]. The functional significance of AMP accumulation in response to EP3 receptor inhibition remains to be elucidated.

The lack of effect of the EP2 receptor blockade on distention-induced release of s-NTDs is intriguing as it suggests that endogenous PGE_2_ differentially regulates the release of s-NTDs in the LP of unfilled and filled bladders. The functional significance of increased eATP degradation in nondistended preparations could be to prevent excessive accumulation of ATP at receptor sites during the early stages of bladder filling and thus avoid the development of bladder hyperactivity. The increased production of adenosine (which inhibits detrusor contractility) further prevents the occurrence of excessive bladder excitability. This mechanism is likely turned off at the end of bladder filling when higher concentrations of ATP are needed to stimulate sensory neurons and/or detrusor smooth muscle cells and initiate voiding.

As discussed, bladder distention increases the production and release of PGs (e.g., PGE_2_, PGF_2α_, and PGI_2_) [5,7,71]. Moreover, increased production and release of PGs or PG receptor activation have been observed in bladder inflammation [67], mechanical trauma of the urothelium [11], interstitial cystitis/bladder pain syndrome [72], overactive bladder [55], and diabetic bladder [73]. In such conditions, prostanoid receptors in cells close to the site of PG release would be exposed to significantly higher PG concentrations than under physiological conditions. It is unknown how high concentrations of different PGs would affect the spontaneous and distention-induced release of s-NTDs. Therefore, we next evaluated the effects of the five PGs applied exogenously at equimolar concentrations (i.e., 10 μM) on the degradation of eATP by s-NTDs released in the LP of unfilled and filled bladders.

Exogenous PGE_2_ increased the constitutive release of s-NTDs and the subsequent degradation of eATP. The increasing effect of exogenous PGE_2_ on eATP degradation was blocked by antagonists of EP3 and EP4 receptors, whereas the effect of endogenous PGE_2_ on the basal release of s-NTDs was mediated by EP2 and EP3 receptors. In other words, EP2 receptors mediated the effects of endogenous but not exogenous PGE_2_. EP3 receptors mediated the effects of both endogenous and exogenous PGE_2_, and EP4 receptors mediated the effects of exogenous but not endogenous PGE_2_. It appears, therefore, that endogenous and exogenous PGE_2_ utilize different mechanisms to increase the basal release of s-NTD and to accelerate the extracellular ATP degradation. The mechanosensitive (in distended bladders) release of s-NTDs was also regulated differently by endogenous and exogenous PGE_2_. While endogenous PGE_2_ had no effect on the s-NTD release, exogenous PGE_2_ increased the release of s-NTDs, activating all four EP prostanoid receptors. This could be due to different concentrations of endogenous and exogenous PGE_2_. As discussed, the basal production and release of PGs in healthy tissues is usually very low [69,74] and likely insufficient to activate the nearby EP receptors, whereas the high concentrations of exogenous PGE_2_ have likely reached and activated the EP receptors present in cells in the LP. Together, these results suggest that (1) the increased release of s-NTDs that is evoked by endogenous and exogenous PGE_2_ (low and high PGE_2_ concentrations) utilizes different EP receptors, and (2) different mechanisms underlie the effects of PGE_2_ on the constitutive and mechanosensitive release of s-NTDs in the LP.

It has been suggested that in addition to EP1-4 prostanoid receptors, PGE_2_ can also activate the FP receptor [24] that is commonly assumed to be targeted primarily by PGF_2α_ [16]. Indeed, the enhancing effect of PGE_2_ on both spontaneous and mechanosensitive s-NTD release was abolished by an FP receptor antagonist, suggesting that PGE_2_ accelerates the ATP degradation activating FP receptors in addition to EP receptors. Exogenous PGF_2α_ increased the degradation of eATP by spontaneously released s-NTDs but not by s-NTDs released during bladder filling. The increasing effect of PGF_2α_ on basal s-NTD release was reduced by the FP receptor antagonist AL8810. It has been proposed that the FP receptor-mediated effects of exogenous PGE_2_ might result from the PGE_2_ conversion to PGF_2α_ upon contact with tissue [24,75]. This is an unlikely scenario in our study, however, because (i) PGE_2_ (the parent compound) had a more pronounced effect on the degradation of eATP than PGF_2α_; (ii) AL8810 blocked the effect of PGE_2_ better than the effect of PGF_2α_; and (iii) PGE_2_, but not PGF_2α_, increased the distention-induced release of s-NTDs via activation of FP receptors. Therefore, PGE_2_ might be a direct ligand of the FP receptor in our system. Exogenous PGF_2α_ might also activate other PG prostanoid receptors and cause mild inhibition of the eATP degradation, which was only revealed in the eADO levels at 30–60 min of reaction.

PGD_2_ is another proinflammatory mediator that is produced in immune cells (e.g., mast cells) and has potential roles in inflammation, angiogenesis, tissue remodeling, fibrosis, bronchospasm, and allergic reactions [76,77]. In the bladder, PGD_2_ has received less attention than PGE_2_, PGF_2α_, and PGI_2_. PGD_2_ is released in the guinea pig bladder [41], and both DP1 and DP2 receptors that are primarily targeted by PGD_2_ are expressed within the bladder wall [40]. PGD_2_ has been shown to inhibit detrusor contractions evoked by nerve stimulation [41]. The DP1 receptor is coupled primarily to the Gα_s_ subunit, whereas the DP2 receptor is coupled to Gα_i_ [16]. However, as pointed out, the DP receptors may couple to other G subunits depending on the cellular context. As discussed, endogenous PGD_2_ did not seem to play a significant role in the degradation of extracellular ATP by s-NTDs. In contrast, exogenous PGD_2_ significantly increased the hydrolysis of eATP by s-NTDs released in the LP of either unfilled or filled preparations. Interestingly, the increasing effects of PGD_2_ on spontaneously released s-NTDs were abolished in the presence of a DP2 but not a DP1 receptor antagonist, while the effect of PGD_2_ on distention-induced release of s-NTDs was inhibited by both DP1 and DP2 receptor antagonists. Therefore, this is another example of differential regulation of s-NTD release in nondistended and distended bladders. Another interesting observation was that simultaneous blockade of DP1 and DP2 receptors with the dual DP1/DP2 antagonist AMG 853 revealed a novel response to exogenous PGD_2_; that is, the usual facilitating effect of PGD_2_ on distention-induced release of s-NTDs was inverted, leading to an inhibition of eATP hydrolysis. It is possible that exogenous PGD_2_ activates additional (e.g., G-protein-independent) signaling pathways [78] that may inhibit the s-NTD release in the distended LP. Further studies are warranted to elucidate the mechanisms underlying the effects of PGD_2_ on the release of s-NTDs in the LP.

PGI_2_ and TXA_2_ appeared to be at odds with PGE_2_, PGD_2_, and PGF_2a_ with regard to ATP degradation in the LP by s-NTDs. Thus, exogenous PGI_2_ and a stable TXA_2_ analog (i.e., U46619) both failed to alter the degradation of eATP by s-NTDs released in EL solutions of unfilled and filled bladder preparations. Increased production of eADO was observed in the presence of an IP receptor antagonist, while the decrease in eATP and the increase in eADP and eAMP remained unchanged. These results suggest that endogenous PGI_2_ might slightly inhibit the release of s-NTDs during bladder filling. However, more studies are needed to confirm such possibilities.

In summary, while all PGs share some common features, including involvement in inflammation, regulation of smooth muscle contractility, and sensory nerve firing, they can differentially influence the release of s-NTDs in the bladder urothelium, thus affecting the levels of extracellular purines during bladder filling and ultimately modifying bladder excitability. Most endogenous PGs did not seem to participate in a significant way in the regulation of ATP hydrolysis by s-NTDs in the non-diseased bladder LP (Table 4 and Table 5).

Endogenous PGE_2_ was an exception as at resting states, it facilitated the release of s-NTDs via EP2 and EP3 receptor activation, likely preventing high accumulation of extracellular ATP at the early stages of bladder filling. Conversely, exogenous PGE_2_ and PGD_2_, and to a lesser extent PGF_2α_, facilitated the release of s-NTDs in the nondistended bladder LP and accelerated the degradation of ATP at rest. The effects of PGE_2_ were mediated by EP3, EP4, and FP receptors, whereas the effects of PGD_2_ and PGF_2α_ were mediated by DP2 and FP receptors, respectively. Exogenous PGE_2_ and PGD_2_ also facilitated the mechanosensitive release of s-NTDs. The effects of PGE_2_ on distention-induced release of s-NTDs were mediated by EP1, EP2, EP3, EP4, and FP receptors, whereas the effects of PGD_2_ were mediated by both DP1 and DP2 receptors. Therefore, PGE_2_ and PGD_2_ had similar end effects and accelerated the degradation of extracellular ATP by s-NTDs that were released either at rest or during bladder filling. This might be a particularly important self-guard mechanism in disease states with increased bladder activity (e.g., inflammation, overactive bladder, and bladder pain syndrome), when high amounts of PGs and ATP are released in the bladder wall. Increased hydrolysis of ATP and formation of adenosine would prevent or impede the development of excessive bladder excitability and activity. PG-mediated control of s-NTD release appeared to be a sophisticated system since different receptor mechanisms mediated the effects of PGs on the constitutive and distention-induced release of s-NTDs and hence on the degradation of ATP at the early and late stages of bladder filling. Likewise, different EP receptors mediated the effects of low (endogenous) and high (exogenous) PG concentrations. Finally, our study demonstrated that additional (and opposite) mechanisms for modulation of s-NTD release could be revealed when both DP1 and DP2 receptors were blocked. PG receptor and signaling pathways could become a target for treating bladder excitability disorders accompanied by elevated extracellular ATP.

It remains to be elucidated how PGs stimulate the release of s-NTDs in the bladder LP. Similarly, it is currently unknown how s-NTDs are released in the extracellular space. It might be that s-NTDs are released through ectodomain shedding of membrane-bound NTDs, a membrane proteinase-mediated posttranslational modification that regulates the functions of hundreds of proteins [79]. Notably, it appears that isoprostanes (isomers of the conventional PGs that are produced in vivo primarily as a result of oxidative stress) [80], as well as some PGs (e.g., PGF_2α_), can stimulate the shedding of membrane proteins by activating shedding-induced metalloproteinases [81]. Future studies are needed to determine if such mechanisms underlie the stimulation of s-NTD release by PGs in the bladder LP.

## 4. Materials and Methods

### 4.1. Animal Model

#### 4.1.1. Euthanasia and Tissue Collection

Twelve- to sixteen-week-old male C57BL/6J mice (Jackson Laboratory, Bar Harbor, MN, USA) were euthanized by sedation with isoflurane (AErrane; Baxter, Deerfield, IL, USA), followed by cervical dislocation. The urinary bladder was removed for subsequent dissection and placed in ice-cold oxygenated Krebs-bicarbonate solution (KBS) with the following composition (mM): 118.5 NaCl, 4.2 KCl, 1.2 MgCl_2_, 23.8 NaHCO_3_, 1.2 KH_2_PO_4_, 11.0 dextrose, and 1.8 CaCl_2_ (pH 7.4).

#### 4.1.2. Ethical Approval

All animals were maintained and experiments were performed following the National Institutes of Health Guide for the Care and Use of Laboratory Animals and the Institutional Animal Use and Care Committee at the University of Nevada, Reno (Protocol #20-9-1077).

### 4.2. Ex Vivo Detrusor-Free Bladder Preparation

Bladder preparations with denuded detrusor smooth muscle were prepared as described in previous works [65,82]. After obtaining the bladder from the animal, it was pinned to a Sylgard dish by the ureters, the urethra, and the serosal layer at the apex. The bladder was maintained in fresh, cold KBS solution with a constant supply of a gaseous solution (95% O_2_, 5% CO_2_) throughout the dissecting process. The connective and fat tissues surrounding the bladder and ureters were removed, and the detrusor was gently cut and removed with surgical scissors, leaving the urothelium exposed and intact. After removing the detrusor, a PE-20 catheter was inserted through the urethra and secured using at least two 6–0 silk and nylon surgical sutures. The approximate bladder volume was determined by manual filling of the bladder using a BD 1 mL syringe filled with KBS. Following that stage, the preparation was placed in a 3 mL custom-made water-jacketed chamber with its bottom covered with Sylgard. The chamber was filled with KBS; kept at 37 °C and pH 7.4; and constantly supplied with 95% O_2_, 5% CO_2_. The catheter was connected to an infusion syringe pump (Genie Touch, Kent Scientific, Torrington, CT, USA) for bladder filling.

### 4.3. Soluble Nucleotidase (s-NTD) Activity in Concentrated Extraluminal Solutions

#### 4.3.1. Collection of Extraluminal Solutions Containing s-NTDs

The detrusor-free bladder preparation was placed in the 3 mL chamber and equilibrated for 20–30 min with vehicle (KBS or DMSO 0.2%) or drug, as required by the experimental protocol. After the equilibration, the solution in the chamber was replaced with a fresh solution, and the bladder was left empty (nondistended condition) for a time equivalent to the time needed to achieve a distended condition (~85–90% of bladder volume estimated at the time of dissection). Next, 2.9 mL of the EL solution in the chamber in contact with the nondistended bladder LP was transferred to a 4 mL Amicon centrifugal tube with a molecular weight cut-off (MWCO) pore size of 10 kDa (Millipore Sigma, Burlington, MA, USA) for centrifugation. Then, a fresh solution containing the drug/vehicle was added to the chamber, and the bladder was filled with KBS at a rate of 15 μL/min to ~85–90% of its maximum capacity (distended condition). At the end of filling, 2.9 mL of ELS was collected and transferred to a centrifugal tube as described above. The ELSs collected from distended and nondistended bladder preparations were processed in identical manners. Bladder preparations were kept in contact with receptor antagonists throughout dissection, equilibration, and bladder filling (or equivalent time for nondistended preparations) to ensure effective receptor inhibition. Receptor agonists were applied only during filling (or equivalent time in nondistended preparations) of the bladder to avoid receptor desensitization. Only one drug was tested in each bladder preparation.

#### 4.3.2. Concentration of Extraluminal Solutions Containing s-NTDs

The Amicon centrifugal tubes containing ELSs of nondistended and distended bladder preparations were placed in a SorvallST 40R centrifuge (Thermofisher, Langenselbold, Germany) and centrifuged at 4000× *g* for 25 min at 4 °C to a final volume of ~100 μL. Once the EL samples were centrifuged, the concentrated ELSs (cELSs) were transferred to 600 μL Eppendorf tubes and adjusted to a final reaction volume of 200 μL with fresh KBS as described previously [33,59,60,61].

#### 4.3.3. Time Course of Extracellular eATP Degradation by s-NTDs in cELS of Nondistended and Distended LP

To evaluate the activity of s-NTDs, the substrate 1,*N^6^*-etheno-adenosine 5′-triphosphate (eATP, 2 μM) (with 1 × 10^6^ times greater fluorescence than regular ATP) [83] was added to the 600 μL reaction tubes containing cELS with s-NTDs. The reaction tubes were kept at 37 °C throughout the time courses of enzymatic reactions. Twenty-microliter aliquots were collected at 10″, 2′, 4′, 6′, 8′, 10′, 20′, 30′, 40′, and 60′ after contact of the enzymes with the substrate and added to 300 μL HPCL inserts containing 180 μL cold citric buffer to stop the enzymatic reaction, preserve the 1,*N^6^*-etheno purines present in the samples, and dilute the samples 10-fold. The samples were preserved at −20 °C until subsequent analysis using HPLC techniques.

### 4.4. HPLC Analysis of 1,N^6^-Etheno-Purines

The decrease in the substrate eATP and the formation of its metabolites, 1,*N^6^*-etheno-adenosine 5′-diphosphate (eADP), 1,*N^6^*-etheno-adenosine 5′-monophosphate (eAMP), and 1,*N^6^*-etheno-adenosine (eADO), were analyzed by a reverse-phased gradient Agilent Technologies 1200 liquid chromatography system equipped with a fluorescence detector (HPLC-FLD) (Agilent Technologies, Wilmington, DE, USA) as described previously [33]. The amounts of 1,*N^6^*-etheno-purines per sample were measured using reference standards that contained known levels of eATP, eADP, eAMP, and eADO.

### 4.5. Drugs

The following drugs were obtained from Tocris (Bio-Techne, Minneapolis, MN, USA): AMG 853, L-798,106, L-161,982, PGE_2_, PGF2α, PGI_2_, PF04418948, RO1138452, SC51322, and U46619. The following drugs were obtained from Cayman Chemical (Ann Arbor, MI, USA): AL8810, PGD_2_, OC000459, and S-5751. The following drugs were obtained from Sigma-Aldrich (St. Louis, MO, USA): ATP, ADP, AMP, adenosine, and dimethyl sulfoxide (DMSO). ATP, ADP, AMP, and adenosine were used to prepare HPLC standards. DMSO 0.2% was used to dissolve drugs. Pilot studies showed that the effects of DMSO 0.2% were identical to the effects of KBS. A list of drugs, used concentrations, and vehicles/solvents is depicted in Table 1.

### 4.6. Statistical Analysis of Data

The area of the chromatogram peaks that corresponded to each 1,*N^6^*-etheno-purine was analyzed with ChemStation v. B04.03 (Agilent Technologies, Wilmington, DE, USA) and plotted against a standard curve. The data were processed using Excel (Microsoft Corporation, Redmond, WA, USA) and the GraphPad Prism v.8.4.3 software (GraphPad Inc., San Diego, CA, USA). Differences between time courses were analyzed by a two-way analysis of variance (ANOVA) with Sidak’s post-test when comparing two groups and by a two-way analysis of variance (ANOVA) with Tukey’s post-test when comparing more than two groups. All values were presented as the mean ± SD. Data were considered statistically significant when the *p* values were <0.05.

Parts of this work were previously presented in abstract form at the Society for Pelvic Research, Savannah, GA, USA, 7–9 December 2023.

## Figures and Tables

**Table 1:** Drugs and concentrations used.

Name	Type	Concentration (μM)	Vehicle
PGE_2_	EP1-4 receptor agonist	10	DMSO
PGD_2_	DP1, DP2 receptor agonist	10	DMSO
PGF_2α_	FP receptor agonist	10	DMSO
PGI2	IP receptor agonist	10	DMSO
U46619	TP (TXA2) receptor agonist	10	DMSO
SC51322	EP1 receptor antagonist	1	DMSO
PF04418948	EP2 receptor antagonist	1	DMSO
L-798,106	EP3 receptor antagonist	0.25	DMSO
L-161,982	EP4 receptor antagonist	1	DMSO
S-5751	DP1 receptor antagonist	1	DMSO
OC000459	DP2 receptor antagonist	10	DMSO
AMG 853	DP1/DP2 receptor antagonist	1	DMSO
AL8810	FP receptor antagonist	10	DMSO
RO1138452	IP receptor antagonist	10	DMSO
Indomethacin	COX1/COX2 inhibitor	10	DMSO

**Table 2:** Degradation of eATP and formation of e-products in EL solutions of nondistended bladder preparations.

	Vehicle (n = 6)	PGI_2_ (n = 4)	RO1138452 (n = 6)	RO1138452 + PGI_2_ (n = 4)	U46619 (n = 4)	Indomethacin (n = 4)
eATP	56.67 ± 5.8	59.69 ± 8.16	54.39 ± 16.58	55.79 ± 10.63	53.63 ± 10.26	57.52 ± 11.21
eADP	33.79 ± 3.79	31.35 ± 5.08	34.71 ± 10.33	33.78 ± 7.12	35.24 ± 5.47	32.40 ± 7.79
eAMP	7.55 ± 1.69	6.39 ± 0.92	8.12 ± 3.62	7.94 ± 2.64	8.40 ± 2.18	9.00 ± 2.66
eADO	1.99 ± 0.62	2.57 ± 2.17	2.77 ± 2.83	2.49 ± 1.02	2.74 ± 2.78	1.08 ± 1.00 ****

Each purine is presented as % of total purines (eATP + eADP + eAMP + eADO) at 60 min of reaction. Values are shown as the means ± SD. n, number of bladders. **** *p* < 0.0001 vs. vehicle (two-way ANOVA with Sidak’s multiple comparisons test).

**Table 3:** Degradation of eATP and formation of e-products in EL solutions of distended bladder preparations.

	Vehicle (n = 6)	PGI_2_ (n = 4)	RO1138452 (n = 6)	RO1138452 + PGI_2_ (n = 4)	U46619 (n = 4)	Indomethacin (n = 4)
eATP	22.53 ± 9.09	27.53 ± 11.58	13.53 ± 9.38	23.32 ± 12.80	19.42 ± 8.00	30.70 ± 13.90
eADP	48.63 ± 2.83	47.41 ± 2.70	45.86 ± 3.37	46.77 ± 2.42	47.46 ± 2.16	46.37 ± 5.84
eAMP	19.33 ± 3.60	15.53 ± 2.17	21.14 ± 5.78	20.25 ± 8.58	21.56 ± 2.98	18.32 ± 4.99
eADO	9.50 ± 4.55	9.23 ± 7.80	19.47 ± 12.32 ***	9.66 ± 5.70	11.56 ± 8.64	4.61 ± 3.33 ****

Each purine is presented as % of total purines (eATP + eADP + eAMP + eADO) at 60 min of reaction. Values are shown as the means ± SD. n, number of bladders. *** *p* < 0.001, **** *p* < 0.0001 vs. vehicle (two-way ANOVA with Sidak’s multiple comparisons test).

**Table 4:** Summary of the effects of endogenous PGs on s-NTD release/ATP hydrolysis.

	PGE_2_	PGF_2α_	PGD_2_	PGI_2_
Nondistended LP	↑ EP2, ↑ EP3	→	→	→
Distended LP	→	→	→	↓ IP

↑ increase. ↓ decrease. → no change.

**Table 5:** Summary of the effects of exogenous PGs on s-NTD release/ATP hydrolysis.

	PGE_2_	PGF_2α_	PGD_2_	PGI_2_	TXA_2_
Nondistended LP	↑ EP3, ↑ EP4, ↑ FP	↑ FP	↑ DP2	→	→
Distended LP	↑ EP1, ↑ EP2, ↑ EP3, ↑ EP4, ↑ FP	→	↑ DP1, ↑ DP2, ↓“X”	→	→

↑ increase. ↓ decrease. → no change. “X”, unknown pathway.

## Data Availability

The raw data supporting the conclusion of this article will be made available by the corresponding author in the Dryad public data repository (DOI: 10.5061/dryad.djh9w0w90).
